# NtbHLH49, a jasmonate-regulated transcription factor, negatively regulates tobacco responses to *Phytophthora nicotianae*

**DOI:** 10.3389/fpls.2022.1073856

**Published:** 2022-12-06

**Authors:** Wenjing Wang, Jianhui Zhang, Yi Cao, Xingyou Yang, Fenglong Wang, Jinguang Yang, Xiaoqiang Wang

**Affiliations:** ^1^ Tobacco Research Institute, Chinese Academy of Agricultural Sciences, Qingdao, China; ^2^ Sichuan Tobacco Science Research Institute, Chengdu, China; ^3^ Academy of Guizhou Tobacco Sciences, Guiyang, China

**Keywords:** *Phytophthora nicotianae*, tobacco, jasmonate, AtBPE-like, NtbHLH49

## Abstract

Tobacco black shank caused by *Phytophthora nicotianae* is a devastating disease that causes huge losses to tobacco production across the world. Investigating the regulatory mechanism of tobacco resistance to *P. nicotianae* is of great importance for tobacco resistance breeding. The jasmonate (JA) signaling pathway plays a pivotal role in modulating plant pathogen resistance, but the mechanism underlying JA-mediated tobacco resistance to *P. nicotianae* remains largely unclear. This work explored the *P. nicotianae* responses of common tobacco cultivar TN90 using plants with RNAi-mediated silencing of *NtCOI1* (encoding the perception protein of JA signal), and identified genes involved in this process by comparative transcriptome analyses. Interestingly, the majority of the differentially expressed bHLH transcription factor genes, whose homologs are correlated with JA-signaling, encode AtBPE-like regulators and were up-regulated in *NtCOI1*-RI plants, implying a negative role in regulating tobacco response to *P. nicotianae*. A subsequent study on NtbHLH49, a member of this group, showed that it’s negatively regulated by JA treatment or *P. nicotianae* infection, and its protein was localized to the nucleus. Furthermore, overexpression of *NtbHLH49* decreased tobacco resistance to *P. nicotianae*, while knockdown of its expression increased the resistance. Manipulation of *NtbHLH49* expression also altered the expression of a set of pathogen resistance genes. This study identified a set of genes correlated with JA-mediated tobacco response to *P. nicotianae*, and revealed the function of AtBPE-like regulator NtbHLH49 in regulating tobacco resistance to this pathogen, providing insights into the JA-mediated tobacco responses to *P. nicotianae*.

## Introduction

Tobacco production is hampered by diverse diseases. Tobacco black shank, caused by the hemibiotrophic pathogen *Phytophthora nicotianae*, is one of the most devastating diseases to tobacco cultivation (Meng et al., 2014; Wang and Jiao, 2019). *P. nicotianae* is a species of *Phytophthora* that belongs to the class Oomycetes in the kingdom Chromista and has over 100 species causing destructive damage to a range of plants (Kroon et al., 2012). *P. nicotianae* is a typical soil-borne disease that can infect tobacco plants at any growth stage and affects a broad variety of plants, including Solanaceae crops, horticultural plants, and fruit trees (Kroon et al., 2012). Infection by *P. nicotianae* could cause a sudden withering of the entire plant, as well as root rot and the formation of black patches on the stem near the ground level (Meng et al., 2014). In recent decades, many chemicals have been employed to control tobacco black shank, but their efficacy is rather limited (Meng et al., 2014). Moreover, the majority of chemical pesticides may cause significant environmental pollution (Alexandrino et al., 2022). Development of *P. nicotianae* resistant tobacco cultivars is still one of the most economical and effective approaches to reduce yield loss caused by this pathogen, therefore, identification of genes associated with this pathogen is of great importance.

Studies have shown that plant resistance to *Phytophthora* pathogens is influenced by environmental variables and phytohormones. For example, abiotic stresses and the induced phytohormone abscisic acid (ABA) may attenuate the severity of disease caused by the *Phytophthora* pathogen in plants (Roubtsova and Bostock, 2009; Dileo et al., 2010). Reducing reactive oxygen species (ROS) and ethylene (ET) in susceptible tobacco plants may increase pathogen resistance to *P. nicotianae* (Wi et al., 2012; Cho et al., 2013). Salicylic acid (SA) plays a positive role in regulating the resistance of potato (*Solanum tuberosum*) plants to *P. infestans* (Halim et al., 2007; Zhou et al., 2018). Furthermore, studies on *Arabidopsis* AtRTP5 showed that this factor could regulate the resistance to *Phytophthora* pathogen by modulating the biosynthesis of both SA and jasmonate (JA), implying that JA is involved in regulating plant resistance to *Phytophthora* (Li et al., 2020). The involvement of JA in tobacco resistance to *Phytophthora* pathogen was also indicated (Long et al., 2021). However, the mechanism underlying JA-mediated tobacco resistance to *P. nicotianae* is still largely unclear.

The JA-signaling pathway plays essential role in modulating plant responses to biotic and abiotic stresses (Howe et al., 2018). Extensive researches suggest that JA-signaling pathway is indispensable for plant resistance to both fungal pathogens (*e.g.*, *Alternaria brassicicola*, *Botrytis cinerea*) and bacterial pathogens (*e.g.*, *Pseudomonas syringae* spp.) (Thomma et al., 1998; Kloek et al., 2001). JA-signaling pathway functions in a complicated crosstalking network with SA, ET, and other phytohormones during the regulation of plant resistance to pathogenic microbes (Pieterse et al., 2012). JA signal is perceived by a multi-component complex consisting of COIl (CORONATINE INSENSITIVE1), JAZ (jasmonate ZIM-domain) protein and an inositol pentyl phosphate molecule (Yan et al., 2009). Perception of JA-Ile (the bioactive derivative of JA) by the receptor complex leads to the degradation of JAZ repressor protein *via* the 26S proteasome pathway, resulting in the release of transcription activators that tune up the downstream JA-responses (Yan et al., 2009). The F-box protein COI1 is a key factor in JA signal transduction. COI1 dysfunction could result in a loss of JA responses (Xie et al., 1998). Studies showed that mutations in COI1 could alter plant resistance to bacterial and fungal pathogens (Kloek et al., 2001; Zhang et al., 2017), and that the JAZ repressor protein is also involved in the regulation of plant resistance to pathogens (Thatcher et al., 2016). Downstream regulators, such as bHLH transcription factors, MYB transcription factors and ERFs (ethylene responsive factors), were found to play important roles in plant resistance to pathogen attack (Müller and Munné-Bosch, 2015; Kazan, 2015; Thatcher et al., 2016; Ren et al., 2020; Ren et al., 2022). Identification the downstream regulators that control tobacco responses to *Phytophthora* pathogen is of great importance for dissecting the function of JA-signaling in *Phytophthora* resistance (Thaler et al., 2004; Li et al., 2020).

In order to discover regulators conducting the JA-mediated *P. nicotianae* resistance, we compared *P. nicotianae* resistance between *NtCOI1-*silenced plants (*NtCOI1-*RI) and control plants, and identified genes involved in JA-mediated responses to this pathogen using comparative transcriptome analysis. A set of genes encoding AtBPE-like bHLH transcription factor were found to be up-regulated in *NtCOI1-*RI plants. Further study on NtbHLH49, one of these regulators, shows that overexpression of *NtbHLH49* decreased tobacco resistance to *P. nicotianae*, while knockdown of this gene increased this resistance. The expression of a set of pathogen resistance genes was regulated by NtbHLH49 during tobacco response to *P. nicotianae*. These findings extend our understanding of plant responses to *Phytophthora* pathogens and provide fundamental molecular information for tobacco resistance breeding against *P. nicotianae.*

## Materials and methods

### Plant materials

Wild type plants of *Nicotiana tabacum* cv. TN90, previously developed *NtCOI1*-silenced plants (*NtCOI1*-RI) and empty-vector-transformed control plants (Wang et al., 2014) were employed for the JA-mediated pathogen resistance assay and gene expression assay of this study. For developing transgenic plants overexpressing *NtbHLH49*, the full-length coding sequence of *NtbHLH49* amplified with primer 5’-CACCATGGATATGGATAGCAAGAATG-3’ and 5’-TATGATCGCCTTACCTGCAACC-3’ was cloned into pENTR-D-TOPO vector (Invitrogen, USA) and then integrated into a binary vector pMDC83-Flag, which was modified from pMDC83-GFP (Curtis and Grossniklaus, 2003), to generate the gene overexpression vector. In order to develop transgenic plants for RNAi-mediated gene silencing of *NtbHLH49*, the 5’-end region of *NtbHLH49* amplified with primer 5’-CACCATGGATATGGATAGCAAGAATG-3’ and 5’-GGGTTCATCATATCACTGAGGT-3’ was cloned into pENTR-D-TOPO vector and integrated into the binary vector pHZPRi-Hyg (Wang et al., 2014) to generate the gene silencing vector. The obtained binary vectors were introduced into *Agrobacterium tumefaciens* LBA4404 to develop transgenic plants as described previously (Wang et al., 2014). Transgenic plants verified to overexpress *NtbHLH49* (*NtbHLH49*-OE) or have *NtbHLH49* silenced (*NtbHLH49*-RI) were utilized for further assays.

For tobacco cultivation, surface-sterilized seeds of tobacco plants were sown on 1/2 MS medium supplemented with 25 mg/L hygromycin, and the resistant seedlings were placed into soil pots and validated by PCR tests. Tobacco plants were cultivated in an indoor growth room at 25°C with a photoperiod of 14 h light/10 h dark. 5-week-old seedlings were used for pathogen challenges, phytohormone treatment and transcriptional assays.

For phytohormone treatment, 5-week-old tobacco seedlings were sprayed with solutions containing 100 μM MeJA (methyl jasmonate) and 0.005% Tween 20. Control plants were sprayed with water containing 0.005% Tween 20. Then, tobacco leaves were detached from plants at the indicated time point for further assays.

### *P. nicotianae* inoculation

The oomycete pathogen *P. nicotianae* (kept in our laboratory) was streaked on oat medium (30 g oatmeal and 20 g agar in 1 L ddH_2_O, pH 7.5) and cultured at 28°C for ten days before inoculating tobacco leaves. Tobacco leaves of each experimental group were divided into two groups, and inoculated with *P. nicotianae* mycelium (4 mm blocks from the growth medium) or mock (medium blocks only), respectively. Three independent replicates were set up for each treatment group. The inoculated leaves were incubated in dark at 28°C. After 72 hours of infection, the disease symptom was inspected. No visible symptom or formation of necrosis spots in leaves indicates resistance to *P. nicotianae*, and the development of water-soaking patches indicates susceptibility to *P. nicotianae*. The number of leaves with necrosis spots, water-soaking patches, or invisible symptoms was surveyed to characterize the *P. nicotianae* resistance of tobacco plants. In addition, a set of these leaves were collected at the indicated time points for transcriptional analyses.

### RNA preparation, transcriptome sequencing and *de novo* assembly

Total RNAs were extracted from tobacco leaf samples using a RN38-EASY SPIN Plus Plant Kit (BioTeke, China) following the manufacturer’s instruction. The RNA quality was assessed using an Agilent 2100 Bioanalyzer (Agilent Technologies, USA). Transcriptome sequencing, *i.e.* next-generation RNA sequencing (RNA-seq), was conducted on an Illumina HiSeq 4000TM platform (Illumina Inc., USA) by Allwegene Technology (Beijing, China) with cDNA libraries constructed for each of the samples. Adapter-related reads, low-quality reads (>50% with quality scores ≤20) and reads containing more than 10% unknown nucleotides were removed from the data to generate reliable clean reads for read assembly. The alignment analysis was performed using TopHat 2.1.1 software. The RNA seq data presented in the study are deposited in the NCBI Sequence Read Archive (SRA) repository, accession number PRJNA901343.

### Analysis of differentially expressed genes and gene annotation

The raw sequencing reads were pre-processed by FastQC (http://cufflinks.cb.umd.edu/). The processed reads were mapped to *Nicotiana tabacum* cv. TN90 genome (https://ftp.ncbi.nlm.nih.gov/genomes/all/GCF/000/715/135/GCF_000715135.1_Ntab-TN90/) and assembled using TopHat 2.1.1 software with the reference annotation. The FPKM (fragments per kilobase of exon model per million mapped reads) values for each gene and the DEGs (differentially expressed genes) were calculated with Cufflinks V2.2.l. The DEGs between *NtCOI1*-RI plants and control plants were determined according to the fold change and q-value.

### qRT-PCR assays

Total RNAs of tobacco leaf samples were prepared as described above. First-strand cDNAs were synthesized using a PrimeScript RT reagent Kit (Takara Bio, Japan). The qRT-PCR amplification was performed in a 25μl reaction. The reaction conditions were as followings: an initial denaturation at 95°C for 1 min; 45 cycles of 95°C for 20 s, 59°C for 20 s and 72°C for 30 s; melting curve analysis between 50-95°C. Three biological replicates were performed for each gene. The relative transcription level of each gene was determined using the 2^-ΔΔCt^ method (Livak and Schmittgen, 2001), with *Actin* gene serving as an internal control. Gene-specific primers used for validation of DEGs, identification of *NtbHLH49*-OE and *NtbHLH49*-RI plants, and determination of the expression of pathogen resistance genes in transgenic plants are listed in **Supplementary Table 1**.

### Subcellular localization assay of NtbHLH49

For determining the subcellular localization of NtbHLH49, its coding sequence was cloned into pENTR-D-TOPO vector as described above. Then, *NtbHLH49* was integrated into pMDC83-GFP vector (Curtis and Grossniklaus, 2003) to generate pMDC83-NtbHLH49-GFP vector for expressing the NtbHLH49-GFP fusion protein, and a modified pMDC83-GFP vector for expressing GFP alone was used as control. The obtained vectors were later introduced into *A. tumefaciens* LBA4404, and introduced into *Nicotiana tabacum* BY-2 cells as described by (Goossens et al., 2003). The subcellular localization of NtbHLH49-GFP and GFP in BY-2 cells was observed under a Leica TCSSP8 confocal microscope.

## Results

### Identification of genes involved in the JA-mediated tobacco resistance to *P. nicotianae*

**Figure 1:**
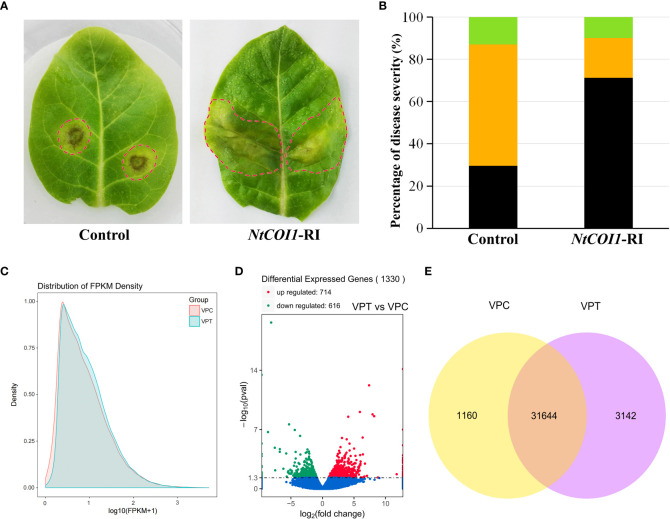
Responses of tobacco plants to *P. nicotianae* infection. **(A)** Representative symptoms of control and *NtCOI1*-RI tobacco leaves infected by *P. nicotianae*. The leaf area with visible symptom was indicated with dashed-circle. **(B)** Percentiles of tobacco leaves with differential symptom severity. The black color indicates water-soaking symptom, the orange color indicates necrosis symptom, and the green color indicates no visible symptom. **(C, D)** The density distribution **(C)**, volcano plot **(D)** and Venn diagram **(E)** of the differentially expressed genes (DEGs) between control and *NtCOI1*-RI plants after *P. nicotianae* infection. VPT indicates *NtCOI1*-RI plants, and VPC indicates control plants.

Plants with RNAi-mediated gene silencing of *NtCOI1*, encoding the JA-Ile receptor protein, were employed to investigate the changes in tobacco responding to *P. nicotianae* upon dysfunction of the JA-signaling. When the leaves of *NtCOI1*-RI plants were infected with *P. nicotianae*, a substantially more severe water-soaking disease symptom was observed in the majority of them after 72 hours incubation at 28°C (**Figure 1A**). On the contrary, the majority of leaves from control plants showed a hypersensitive reaction, with necrosis lesions forming around the *P. nicotianae* infection sites (**Figure 1A**). A quantitative assessment of the severity of the diseases revealed that less than 30% of the leaves of the control plants developed water-soaking patches, but nearly 70% of the leaves of *NtCOI1*-RI plants formed water-soaking spots (**Figure 1B**). Around 60% of the leaves of control plants had necrosis lesions, but only about 20% of the leaves of *NtCOI1*-RI plants had necrosis lesions (**Figure 1B**). The number of leaves with no visible symptom was slightly greater in control plant than in *NtCOI1*-RI plants (**Figure 1B**). These findings support a positive role for NtCOI1 and the JA-signaling pathway in controlling tobacco resistance to this pathogen.

In order to elucidate the mechanism underlying JA/NtCOI1-mediated tobacco resistance to *P. nicotianae*, the infected leaf samples from *NtCOI1*-RI plants and control plants were subjected to a high-throughout RNA-seq assay on an Illumina second-generation high-throughput sequencing platform using the PE150 sequencing strategy. The overall number of mapped reads for control plants is 160,863,066, including 3,934,116 multiple mapped reads and 156,928,950 uniquely mapped reads, and the total number of mapped reads for *NtCOI1*-RI plants is 141,680,162 including 3,141,296 multiple mapped reads and 138,538,866 uniquely mapped reads (**Table 1**; **Supplementary Table 2**). The expression levels of DEGs (differentially expressed genes) were calculated based on the number of uniquely mapped reads using the FPKM (fragments per kilobase of exon model per million mapped reads) method. Among these DEGs, 31,644 genes were expressed in both *NtCOI1*-RI plants and control plants, whereas 1,160 genes and 3,142 genes were specifically expressed in *NtCOI1*-RI plants and control plants, respectively (mean FPKM value>1.0). In the *NtCOI1*-RI plants, 616 genes were found to be down-regulated and 714 genes to be up-regulated. The density distribution, volcano plot and Venn diagram of the FPKM were shown in **Figures 1C–E** to visually exhibit the gene expression profiles between *NtCOI1*-RI plants and control plants. In order to verify the reliability of the gene expression data obtained from the RNA-seq, 9 genes with differential expression levels between *NtCOI1*-RI plants and control plants were chosen and subjected to a qRT-PCR validation assay, which suggested that the gene expression data obtained by RNA-seq are valid for further analysis (**Supplementary Table 3**; **Supplementary Figure 1**).

**Table 1:** Summary of clean reads mapped to the reference genome of tobacco cv. TN90.

Sample	VPC1	VPC2	VPC3	VPT1	VPT2	VPT3
Total mapped	64230840	48962736	47669490	59952524	43282312	38445326
Multiple mapped	1557622	1323320	1053174	1082890	1106880	951526
Uniquely mapped	62673218	47639416	46616316	58869634	42175432	37493800
Read-1	31336609	23819708	23308158	29434817	21087716	18746900
Read-2	31336609	23819708	23308158	29434817	21087716	18746900
Reads map to ‘+’	31336609	23819708	23308158	29434817	21087716	18746900
Reads map to ‘-’	31336609	23819708	23308158	29434817	21087716	18746900
Non-splice reads	42488919	32174824	29728016	36767505	27515879	24930263
Splice reads	20184299	15464592	16888300	22102129	14659553	12563537

VPT indicates NtCOI1-RI plants and VPC indicates control plants.

### Analysis of DEGs related to *P. nicotianae* resistance

**Figure 2:**
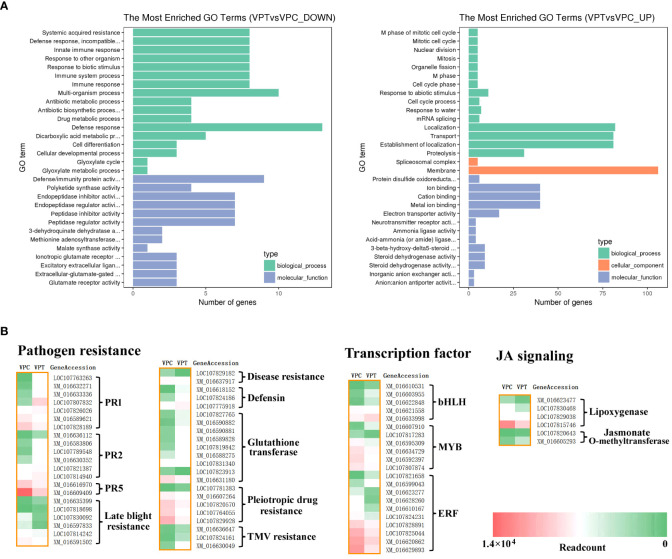
Analysis of DEGs between control and *NtCOI1*-RI plants. **(A)** The enrichment of down-regulated (left) and up-regulated (right) DEGs in *NtCOI1*-RI plants compared with control by GO terms. **(B)** Diagrammatic profile of the differentially expressed pathogen resistance gens, transcription factor genes, and JA-signaling genes in control and *NtCOI1*-RI plants. Color-bar at bottom-right corner indicates the number of readcounts. VPT indicates *NtCOI1*-RI plants and VPC indicates control plants.

A total of 1082 DEGs were annotated in the GO (gene ontology) database using GOseq software (Young et al., 2010), and the GO terms were classified into three ontologies, including biological process, molecular function and cellular component. There were 877 up-regulated and 768 down-regulated DEGs (*NtCOI1*-RI plants vs. Control plants) in the enrichment analysis by GO terms. The GO terms enriched from down-regulated DEGs fell into the categories of systemic acquired resistance, defense response, incompatible interaction, immune response, innate immune response, immune system process, response to other organism, response to biotic stimulus, defense response, defense/immunity protein activity, signaling receptor activity, hormone activity, pathogenesis (**Figure 2A**; **Table 2**; **Supplementary Table 4**). These pieces of information are consistent with the decreased *P. nicotianae* resistance of *NtCOI1*-RI plants. On the other hand, the GO terms enriched from up-regulated DEGs comprise only a few abiotic-responsive DEGs, and the majority of them are engaged in mitotic cell cycle, transportation and other biological processes that have little relevance with pathogen responses (**Figure 2A**).

**Table 2:** Representative GO-enrichment of the down-regulated DEGs (*NtCOI1*-RI vs. control).

GO_accession	Description	Term_type	Over_represented_pValue	Corrected_pValue	DEG_item	DEG_list
GO:0009627	Systemic acquired resistance	Biological_process	3.96E-10	3.32E-07	8	319
GO:0009814	Defense response, incompatible interaction	Biological_process	3.96E-10	3.32E-07	8	319
GO:0045087	Innate immune response	Biological_process	3.96E-10	3.32E-07	8	319
GO:0051707	Response to other organism	Biological_process	1.29E-09	8.13E-07	8	319
GO:0009607	Response to biotic stimulus	Biological_process	1.95E-08	9.83E-06	8	319
GO:0003793	Defense/immunity protein activity	Molecular_function	1.09E-07	4.58E-05	9	319
GO:0002376	Immune system process	Biological_process	0.00013	0.040771	8	319
GO:0006955	Immune response	Biological_process	0.00013	0.040771	8	319

Further analysis was carried out to mine DEGs corresponding to pathogen resistance genes, transcription regulatory genes, and JA-signaling related genes relevant to pathogen resistance. The pathogen resistance genes included *PR* (pathogenesis-related) genes, glutathione transferase genes, late blight resistance genes, pleiotropic drug resistance genes, and TMV resistance genes (**Figure 2B**). The groups that have the majority of genes up-regulated in *NtCOI1*-RI plants comprise *PR1* genes, *PR2* genes (encoding glucanase that cleavages β-1,3-glucans present in fungal cell wall), TMV (tobacco mosaic virus)-resistance genes, defensin genes, and glutathione transferase genes (**Figure 2B**). The groups that have most genes down-regulated in *NtCOI1*-RI plants include *PR5* (osmotin) genes, late blight resistance genes, disease resistance genes, and pleiotropic drug resistance genes (**Figure 2B**). Among the differentially expressed transcription regulatory genes, 5 are bHLH transcription factor genes, 6 are MYB transcription factor genes, and 10 are ERF genes. Furthermore, the identified DEGs also contain a couple of genes engaged in the production or perception of JA (**Figure 2B**), such as genes encoding lipoxygenase (α-linolenic acid metabolism) and jasmonate O-methyltransferase. These findings indicated a sophisticated gene regulation during the JA-mediated *P. nicotianae* responses.

### Phylogenetic analysis of the AtBPE-like regulators with altered expression in *NtCOI1*-RI plants

**Figure 3:**
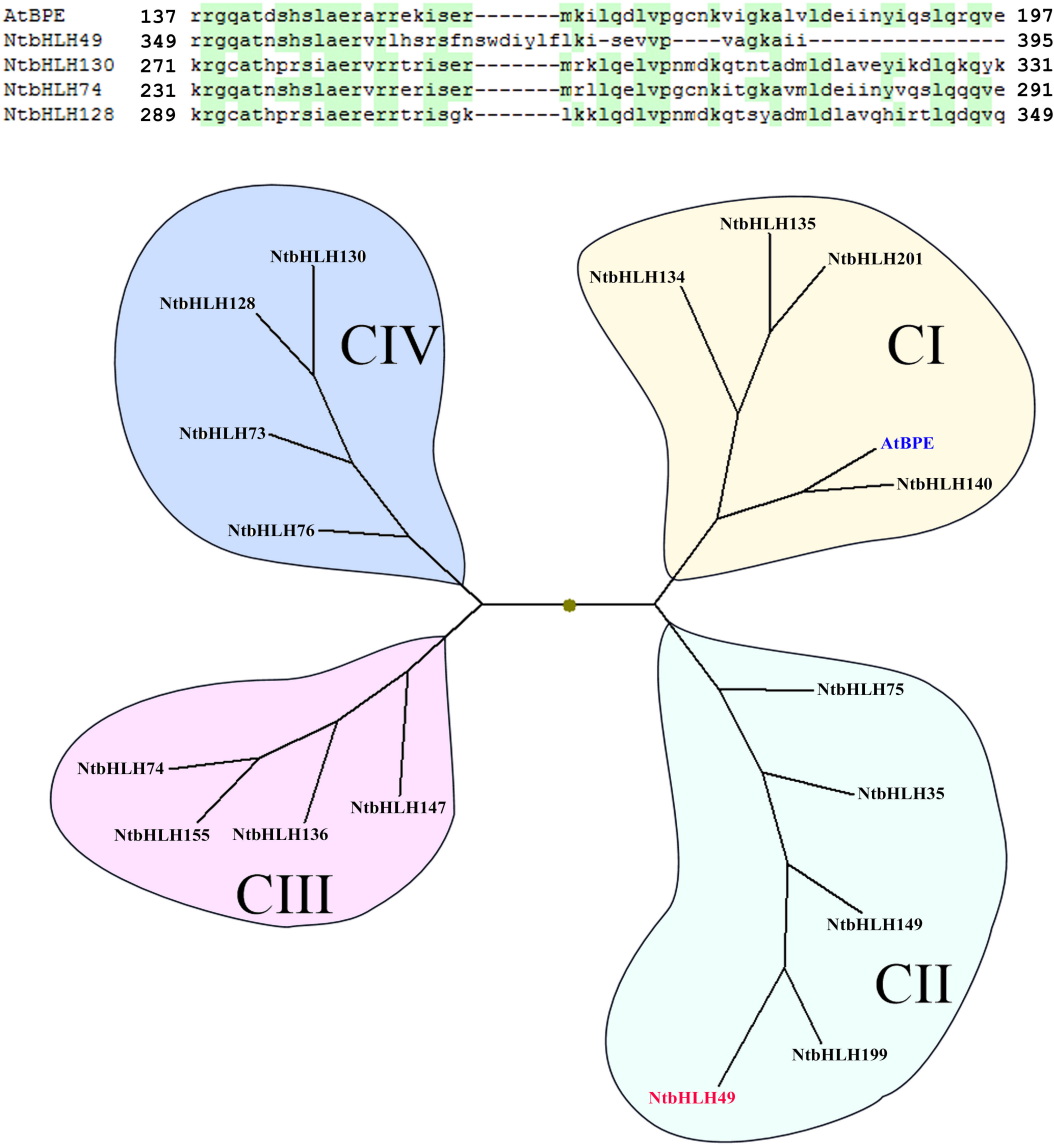
Sequence alignment **(A)** and phylogenetic analyses **(B)** of tobacco AtBPE-like transcription factors. GenBank accession for AtBPE (in blue) is NP_564749, and those for tobacco bHLHs are as followings: NtbHLH49 (in red; XM_016610531), NtbHLH130 (XM_016622848), NtbHLH74 (XM_016633998), NtbHLH128 (XM_016621558), NtbHLH201 (XP_016471633), NtbHLH199 (XP_016500166), NtbHLH134 (XP_016477426), NtbHLH135 (XP_016443360), NtbHLH136 (XP_016462422), NtbHLH140 (XP_016494316), NtbHLH147 (XP_016441121), NtbHLH149 (XP_016478982), NtbHLH155 (XP_016462240), NtbHLH35 (NP_001312938), NtbHLH73 (XP_016497820), NtbHLH75 (XP_016438666), NtbHLH76 (XP_016447869).

As described above, the expression of 5 bHLH genes was found to be altered by the silencing of *NtCOI1*. Interestingly, 4 of them are AtBPE-like regulators belonging to the C-group bHLHs of tobacco (Rushton et al., 2008; **Figures 2C**, **3A**). In order to analyze the phylogenetic relationship of these regulators (i.e., NtbHLH49/130/74/128) to other C-group bHLH transcription factors, the bHLH domains of a set of representative bHLHs were retrieved for the genomic data (Rushton et al., 2008) and subjected to a phylogenetic analysis. As shown in **Figure 3B**, tobacco AtBPE-like regulators could be classified into four subgroups including CI, CII, CIII, and CIV. While AtBPE is close to the CI subgroup members, NtbHLH49 belongs to CII subgroup, NtbHLH74 belongs to CIII subgroup, and NtbHLH130/128 belong to CIV subgroup. The sequence alignment suggested that NtbHLH130/74/128 had a complete bHLH domain that was highly similar to that of AtBPE, however, NtbHLH49 had only a partial bHLH domain (**Figure 3B**). These findings cued us to perform further studies to explore the roles of NtbHLH49 in regulating tobacco responses to *P. nicotianae* attack.

### Expression pattern and subcellular localization of NtbHLH49

**Figure 4:**
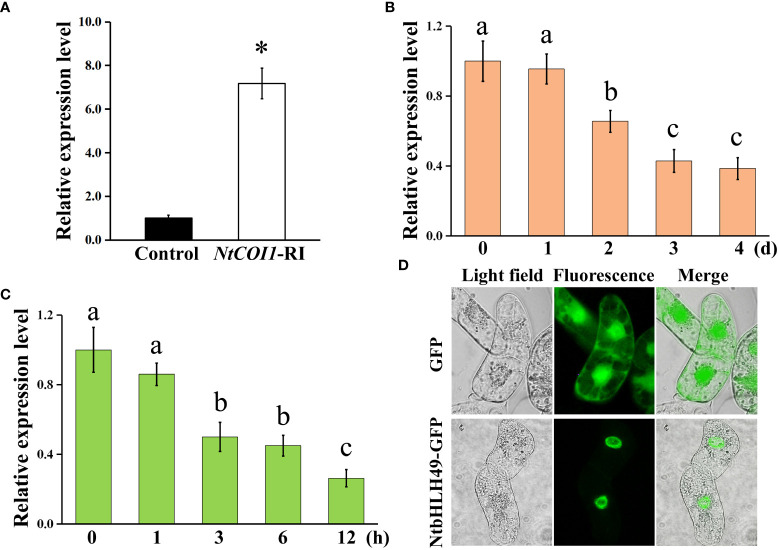
Gene expression pattern and subcellular localization of NtbHLH49. **(A)** Relative expression of *NtbHLH49* in control and *NtCOI1*-RI plants. **(B)** Relative expression of *NtbHLH49* in tobacco leaves after different time of *P. nicotianae* infection. **(C)** Relative expression of *NtbHLH49* in tobacco leaves after different time of MeJA treatment. *Actin* gene was used as an internal control. The expression level of *NtbHLH49* in control plants **(A)** or the time point ‘0’ **(B, C)** was set as “1”. Data are shown as means ± SD (n = 3). Asterisk in **(A)** indicates significant difference from control (Student's t-test; P<0.05). Lower-case letters (i.e., a, b, and c) in **(B–C)** indicate significant difference among the values (p<0.05). **(D)** Subcellular localization of NtbHLH49-GFP fusion protein and GFP alone (control) in BY2 cells.

In order to determine the expression pattern of *NtbHLH49* in tobacco, we first analyzed its expression in the leaves of *NtCOI1*-RI plants and control plants. The results showed that the expression level of *NtbHLH49* in *NtCOI1*-RI plants was about 6 folds of that in control plants (**Figure 4A**), confirming that its expression is negatively regulated by the JA signal perception protein NtCOI1. Then, the expression of *NtbHLH49* responding to *P. nicotianae* attack and MeJA treatment was determined. Two days after the inoculation of *P. nicotianae*, the expression of *NtbHLH49* was suppressed and it was suppressed by over 50% after 3 d of infection (**Figure 4B**). Upon MeJA treatment, the expression of *NtbHLH49* began to decrease after 1 h of treatment, attenuated by nearly 50% after 3 h of treatment, and decreased by about 70% after 12 h of treatment (**Figure 4C**). These data suggest that the expression of *NtbHLH49* is negatively regulated by *P. nicotianae* attack or MeJA treatment, which indicate that NtbHLH49 may be a negative regulator of tobacco in responding to *P. nicotianae*.

Subsequently, the subcellular localization of NtbHLH49 was investigated by developing transgenic BY-2 cell lines that expressing GFP (yellow fluorescent protein; as control) and the C-terminal GFP fusion of NtbHLH49. As shown in **Figure 4D**, the fluorescence of GFP protein was observed in the cytoplasm and nucleus, while that of the fusion protein NtbHLH49-GFP was only detected in the nucleus. These results showed that NtbHLH49 is a nucleus-localized transcription factor.

### Alteration of *NtbHLH49* expression changed tobacco resistance to *P. nicotianae*

**Figure 5:**
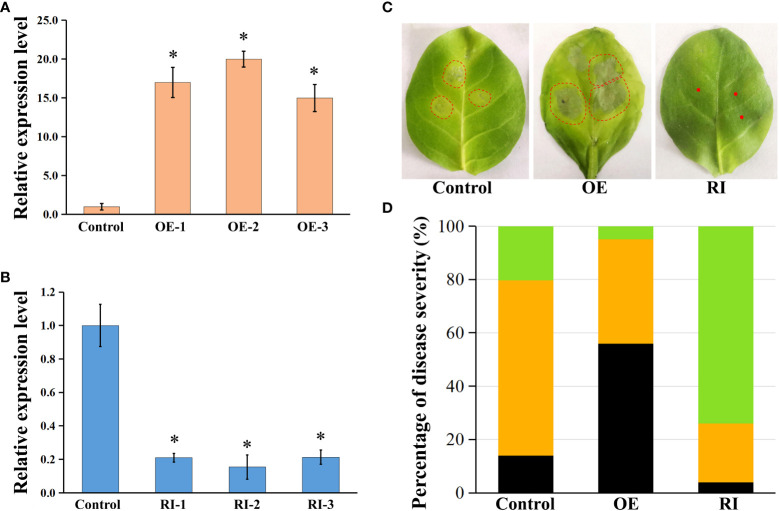
Roles of NtbHLH49 in regulating tobacco resistance to *P. nicotianae*. **(A, B)** Relative expression of *NtbHLH49* in *NtbHLH49*-OE and *NtbHLH49*-RI plants. Tobacco *Actin* gene was used as an internal control. The expression level of *NtbHLH49* in control plant was set as “1”. OE-1/2/3 and RI-1/2/3 indicate different transgenic lines. Data are shown as means ± SD (n = 3). Asterisks indicate significant difference from control (Student's t-test; P<0.05). **(C)** Representative symptoms of control, *NtbHLH49*-OE and *NtbHLH49*-RI tobacco leaves infected by *P. nicotianae*. The leaf area with visible symptom was indicated with dashed-circle. **(D)** Percentiles of control, *NtbHLH49*-OE and *NtbHLH49*-RI tobacco leaves with differential symptom severity. The black color indicates water-soaking symptom, the orange color indicates necrosis symptom, and the green color indicates no visible symptom.

Transgenic tobacco plants overexpressing *NtbHLH49* (*NtbHLH49*-OE) or having *NtbHLH49* knocked down by RNAi-mediated gene silencing (*NtbHLH49*-RI) were developed to further investigate the roles of NtbHLH49 in regulating tobacco resistance to *P. nicotianae*. Three lines that had *NtbHLH49* overexpressed more than 10 folds and another three lines that had the expression of *NtbHLH49* suppressed by near 80% percent were employed for further gene function analyses (**Figures 5A, B**). After infection with *P. nicotianae*, most leaves from control plants formed small necrosis lesions around the inoculation sites, but the leaves from *NtbHLH49*-OE plants developed much severe water-soaking disease symptom around the inoculation sites (**Figure 5C**). In contrast, most leaves from the *NtbHLH49*-RI plants had near invisible necrosis lesions around the inoculation sites, showing an increased resistance to *P. nicotianae*. Quantification of the severity of the diseases showed that control plants had less than 20% of leaves developed water-soaking patches, about 60% of leaves formed necrosis lesions and near 20% of leaves without visible symptom, while *NtbHLH49*-OE plants had over 50% of leaves developed water-soaking spots, near 40% of leaves formed necrosis lesions and less than 10% of leaves without visible symptom (**Figure 5D**). The *NtbHLH49*-RI plants had around 70% of leaves without visible symptom, around 20% of leaves formed necrosis lesions and less than 5% of leaves had water-soaking patches (**Figure 5D**). These findings suggest that NtbHLH49 plays a negative role in controlling tobacco resistance to *P. nicotianae*.

### NtbHLH49 regulated the expression of genes correlated with *P. nicotianae* resistance

**Figure 6:**
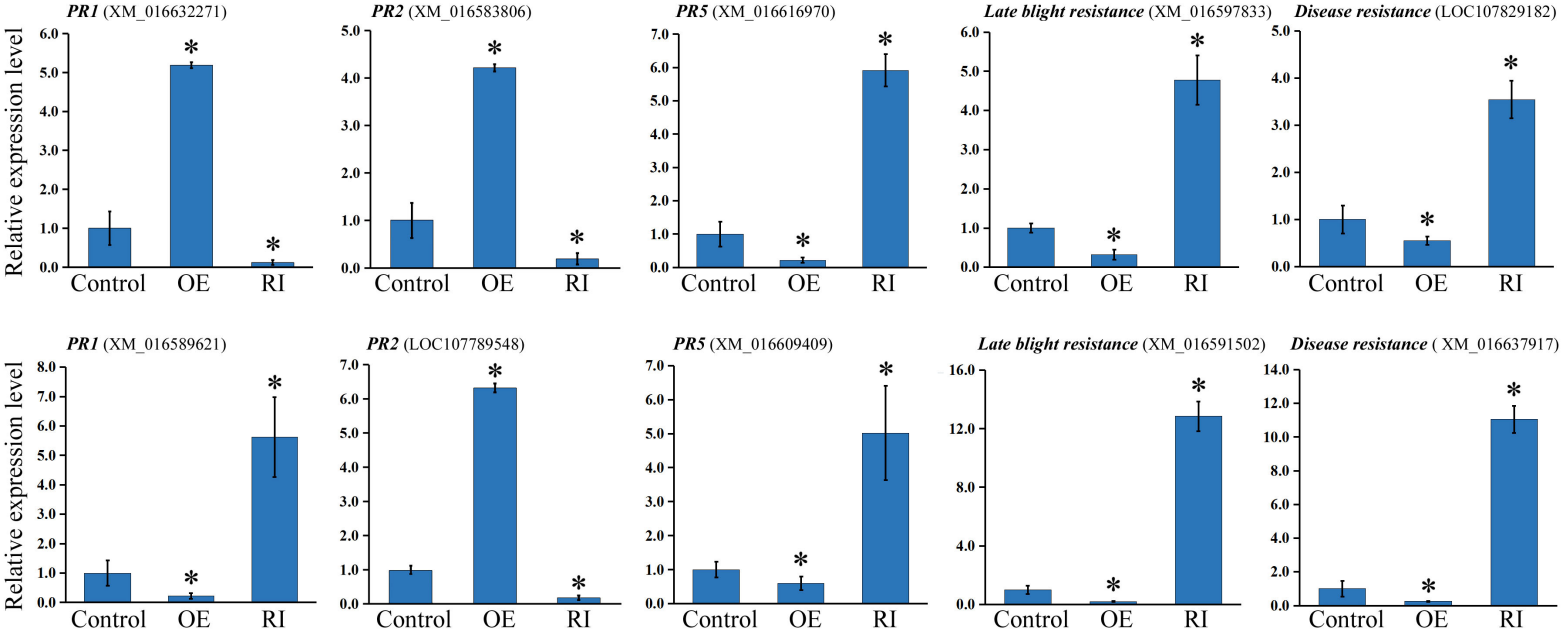
Relative expression of pathogen resistance genes in control, *NtbHLH49*-OE (OE) and *NtbHLH49*-RI (RI) plants. Tobacco *Actin* gene was used as an internal control. The expression level of each gene in control plant was set as “1”. Each value is the average (±SD) of three independent transgenic lines. Asterisks indicate significant difference from control (Student’s *t*-test; *P* < 0.05).

The expression of pathogen resistance genes were inspected to reveal the mechanism underlying NtbHLH49-mediated tobacco resistance to *P. nicotianae*. These tested genes included two *PR1* genes, two *PR2* genes, two *PR5* genes, two late blight resistance genes, and two disease resistance genes. The results showed that one *PR1* gene (GenBank accession: XM_016632271) was increased 5 fold in the *NtbHLH49*-OE plants but was suppressed in the *NtbHLH49*-RI lines, and that the other *PR1* gene (GenBank accession: XM_016589621) was suppressed in the *NtbHLH49*-OE plants but was accentuated over 5 folds in the *NtbHLH49*-RI lines (**Figure 6**), suggesting that NtbHLH49 displayed differential regulation on the expression of specific *PR1* genes. Both of the *PR2* genes (GenBank accession: XM_016583806, LOC107789548) were increased in the *NtbHLH49*-OE plants and were suppressed in the *NtbHLH49*-RI plants (**Figure 6**), showing a positive regulatory of NtbHLH49 on their expression. On the contrary, both the *PR5* genes (GenBank accession: XM_016616970, XM_016609409) were decreased in the *NtbHLH49*-OE plants and were increased in the *NtbHLH49*-RI plants (**Figure 6**), indicating a negative regulatory role on the tested *PR5* genes. Furthermore, NtbHLH49 showed a negative regulatory role on the expression of the two tested late blight resistance genes (GenBank accession: XM_016597833, XM_016591502), and also negatively regulated the expression of the two disease resistance genes (GenBank accession: LOC107829182, XM_016637917) as shown in **Figure 6**. The differential regulation of the tested pathogen resistance genes indicated a complicated role of NtbHLH49 in mediating tobacco resistance to *P. nicotianae*.

## Discussion

Plant resistance to pathogen attack is regulated by multiple phytohormones, such as JA, ET, SA, ABA etc. (Robert-Seilaniantz et al., 2011; Mishra et al., 2020). The JA-/ET-signaling pathway mediates defense against necrotrophic or hemibiotrophic infections (Robert-Seilaniantz et al., 2011). Available studies suggest that plant resistance to *Phytophthora* pathogens is regulated by environmental stresses and phytohormones (Dong et al., 2015; Shibata et al., 2016; Yang et al., 2019). For instance, *P. sojae* RXLR effector PsAvh238 suppresses plant defenses against *P. sojae* by targeting GmACSs, an enzyme that catalyzes ACC formation during ET biosynthesis (Yang et al., 2019). The expression of GmCYP82A3, a soybean CYP82 gene functioning in JA/ET-meidated defensive responses, enhanced *N. benthamiana* resistance to *P. parasitica* (Yan et al., 2016). Additionally, *Arabidopsis* resistance to *Phytophthora* pathogen is modulated by both the SA and JA signaling pathways (Li et al., 2020). These pieces of evidence also indicated the importance of JA-signaling in plant responses to *Phytophthora* pathogen. Utilizing previously developed *NtCOI1*-silenced plants, this work investigated the roles of JA-signaling pathway in mediating tobacco responses to *P. nicotianae* and analyzed the correlated transcriptional regulations using a transcriptome-sequencing approach.

The transcriptome analysis identified a total of 1330 DEGs between *NtCOI1*-RI plants and control plants infected with *P. nicotiane*. 1082 of these DEGs were annotated in the GO database, with 877 being up-regulated and 768 being down-regulated in *NtCOI1*-RI plants. The GO annotation revealed that a large number of pathogen resistance genes, such as those involved in systemic acquired resistance, immune/defense response, defense/immunity protein, biotic stimulus response, pathogenesis, and phytohormone-signaling related, were down-regulated in *NtCOI1*-RI plants. The analysis identified some pathogen resistance genes, transcription regulator genes and phytohormone-signaling related genes. These findings showed that the JA-signaling pathway could manipulate both functional genes and transcription regulatory genes during tobacco responding to *P. nicotiane* attack.

Extensive studies showed that dysfunction of JA-signal perception protein COI1 could alter plant resistance to both bacterial and fungal pathogens (Kloek et al., 2001; Zhang et al., 2017), and that the downstream regulators, such as bHLH transcription factors and MYB transcription factors, played important roles in the JA-mediated plant resistance to pathogen attack (Thatcher et al., 2016; Ren et al., 2020; Ren et al., 2022). The differentially expressed transcription factor genes included a set of bHLH transcription factor genes, MYB transcription factor genes, and ERF genes that may play roles in transducing JA signals and regulating JA-mediated pathogen responses (Solano et al., 1998; Lorenzo et al., 2003). bHLH transcription factors could regulate plant resistance to multiple pathogens, such as *Botrytis cinerea* and *Sclerotinia sclerotiorum* in Arabidopsis and so on (Kazan and Manners, 2013; Li et al., 2018; Teng et al., 2021). They have also been indicated in the regulation of plant *Phytophthora* pathogen resistance. In soybean, the bHLH transcription factor GmPIB1 enhances resistance to *P. sojae* and reduces reactive oxygen species accumulation (Cheng et al., 2018). Potato (*Solanum tuberosum*) bHLH transcription factor *StCHL1* and its *N. benthamiana* homolog could enhance the leaf colonization by *P. infestans* and suppress cell immunity (Turnbull et al., 2017). Genome-wide transcriptome of *N. benthamiana* also suggested that bHLH genes may play important roles in *Phytophthora* pathogen resistance (Yu et al., 2019). Employing common tobacco cultivar TN90, this study revealed that 5 bHLHs transcription factor genes were differentially expressed between *NtCOI1*-silenced plants and control. Intriguingly, the majority of these bHLH transcription factor genes were AtBPE-like regulators and were up-regulated in *NtCOI1*-RI plants, which suggested a negative role in regulating tobacco resistance to *P. nicotianae*. Further study on NtbHLH49, one of these bHLH transcription factor genes, found that its expression was negatively regulated by JA treatment or *P. nicotianae* infection, and that its overexpression decreased and its gene knockdown increased tobacco resistance to *P. nicotianae*. Therefore, these findings suggested that NtbHLH49 is a negative regulator during tobacco responding to *P. nicotianae*. Subsequent transcription assays demonstrated that NtbHLH49 regulated the expression of a set of pathogen resistance genes including *PR1*, *PR2*, *PR5*, and late blight resistance genes etc., and that it exhibited differential regulation on the specific homolog of these pathogen resistance genes, showing a complicated regulatory function of NtbHLH49 in tobacco. Taken together, these assays revealed the function of AtBPE-like regulator NtbHLH49 in regulating tobacco resistance to *P. nicotianae*, and extended the knowledge of bHLH transcription factors in mediating tobacco responses to *P. nicotianae*.

## Conclusion

Using physiological experiments and a high-throughout sequencing approach, this study identified the functional and regulatory genes that may be involved in the JA-mediated *P. nicotianae* responses of tobacco. The results revealed that a set of pathogen resistance genes, transcription factor genes, and JA-signaling genes took part in tobacco resistance to this pathogen, which also indicated the involvement of a group of AtBPE-like regulators in this process. Subsequent studies on NtbHLH49 showed that its gene expression was negatively regulated by JA treatment or *P. nicotianae* infection, and it played a negative role in regulating tobacco resistance to *P. nicotianae*. Findings of this study provide insights into the regulatory roles of AtBPE-like bHLH transcription factors in tobacco responses to *P. nicotianae* and are helpful to unravel the underlying mechanism for tobacco resistance against this pathogen.

## Data availability statement

The names of the repository/repositories and accession number(s) can be found at NCBI, PRJNA901343.

## Author contributions

WW and JZ conceived the study and designed the experiments. WW, JZ, YC, and XY performed the experiments, and produced the figures. All authors analyzed the data. WW and JZ wrote the manuscript, and FW, JY, and XW helped in the manuscript revision. All authors contributed to the article and approved the submitted version.
