# Supplementary material for: NtbHLH49, a jasmonate-regulated transcription factor, negatively regulates tobacco responses to Phytophthora nicotianae
Source: Front Plant Sci. 2022 Dec 6;13:1073856. doi: 10.3389/fpls.2022.1073856 (PMC9764443; doi:10.3389/fpls.2022.1073856)
Supplement: Supplementary file 1 [file Table_1.docx]

**Supplementary Table 1.** qRT-PCR primers for validation of DEGs, identification of transgenic plants, and determination of the expression of pathogen resistance genes.

| **Gene name** | **NCBI gene accession** | **Forward primer** | **Forward primer** | **Note** |
| --- | --- | --- | --- | --- |
| gene69077 | LOC107807832 | 5’-AGCTCAGGGAAGTGGCGATT-3’ | 5’-GCACATCCAACACGAACCGA3’ | Transcriptome data validation by qRT-PCR. |
| gene526 | XM_016610531 | 5’-GGACTAGCGCCAATGTGCAG-3’ | 5’-TGACGGCCACTACAGTCAGC-3’ |  |
| gene14971 | LOC107821658 | 5’-GCTCGGCACTTATCCAACCC-3’ | 5’-TGCAGCAGCAGCTTGAACAT-3’ |  |
| gene61090 | XM_016621994 | 5’-TTTCGGGCACCCGATTCGAT-3’ | 5’-TGGCCTCTCTTGTGGGAACG-3’ |  |
| gene51414 | XM_016609893 | 5’-TCCGTACGTGGTTGCACTCT-3’ | 5’-AGCACCAAATCCACCCACCA-3’ |  |
| gene32393 | LOC107767069 | 5’-CGCTGGTACGAGGTCCAGTC-3’ | 5’-GCTCCCTCAGCTGTTCCCAA-3’ |  |
| gene65961 | XM_016628260 | 5’-AGGCGTAGCAAGACACCACA-3’ | 5’-AGTTTGGTTGGAGGGTCGCA-3’ |  |
| Novel01724 | X13777 | 5’-GGCTCTTTACAGGCCCACGA-3’ | 5’-GCCACGTCCTCTTCCTCCTC-3’ |  |
| gene40818 | LOC107776401 | 5’-ACCGATCAGTCACGACGCTT-3’ | 5’-TTCTGCGATTTGGGCCAACG-3’ |  |
| NtbHLH49 | XM_016610531 | Vector primer:  5’-GCATTCTACTTCTATTGCAGC-3’ | Gene specific primer:  5’-AGGGCCTGGGCCAATTTCTGC-3’ | Transgenic plant identification by qRT-PCR |
| PR1 | XM_016632271 | 5’-CGTGCAGATGTAGCCGTGGA-3’ | 5’-CCGAGTTACGCCAAACCACCT-3’ | qRT-PCR for pathogen resistance related gene. |
|  | XM_016589621 | 5’-ACAAGGAGATGGTGGAGCTGGA-3’ | 5’-TGAAGGAGATGAGATTCCACAGCTT-3’ |  |
| PR2 | XM_016583806 | 5’-CTCCAGCAGATGTCGTGGCT-3’ | 5’-TTGGAGCCTCTGAGGGCTTG-3’ |  |
|  | LOC107789548 | 5’-AGCAAACACCTACCCACCCA-3’ | 5’-TCAGAAGGCCAGCCACTTTCA-3’ |  |
| PR5 | XM_016616970 | 5’-GCGAGGTCAAAGCTGGTGGT-3’ | 5’-CCAGGCTTAGTCGGGCCAAA-3’ |  |
|  | XM_016609409 | 5’-TCCGGCGTATTTGAGGTCCA-3’ | 5’-GGCCCAGAACCACCAGCTTT-3’ |  |
| Late blight resistance | XM_016597833 | 5’-TGCGAGACTGCCCAACCTTC-3’ | 5’-AAGTTGACGGCTGCCCAACA-3’ |  |
|  | XM_016591502 | 5’-CATTGCTCAAGGGCGTCGAT-3’ | 5’-GGTGCTGACCCTTCTCCGAA-3’ |  |
| Disease resistance | LOC107829182 | 5’-TCAGCCAAGAACGTGTGCCA-3’ | 5’-CGTCGCATGGTGTCCGGTTT-3’ |  |
|  | XM_016637917 | 5’-TTGGGCGGAGGAAGTGCAAG-3’ | 5’-GAGAATGGCGGGACCAGCTC-3’ |  |
| Actin | X63603 | 5’-ACTGAAGCACCTCTTAACCCG-3’ | 5’-GTGGCTAACACCATCACCAG-3’ | Internal control for qRT-PCR. |
