# Supplementary material for: NtbHLH49, a jasmonate-regulated transcription factor, negatively regulates tobacco responses to Phytophthora nicotianae
Source: Front Plant Sci. 2022 Dec 6;13:1073856. doi: 10.3389/fpls.2022.1073856 (PMC9764443; doi:10.3389/fpls.2022.1073856)
Supplement: Supplementary file 3 [file Table_3.docx]

**Supplementary Table 3.** Abundance of genes selected for qRT-PCR validation in control (VPC) and *NtCOI1*-RI (VPT) plants.

| **Validated genes** | **Reads in VPC** | **Reads in VPT** | **Log_2__FoldChange (VPT** **vs. VPC)** |
| --- | --- | --- | --- |
| gene69077 | 28.87004827 | 3081.573351 | 6.7379 |
| gene526 | 1.796896311 | 16.00339872 | 3.1548 |
| gene14971 | 2.695344467 | 44.29905718 | 4.0387 |
| gene61090 | 0.299482719 | 14.01685196 | 5.5485 |
| gene51414 | 64.89288503 | 1162.992572 | 4.1636 |
| gene32393 | 2.09637903 | 125.2136981 | 5.9003 |
| gene65961 | 91.13168626 | 13.43196073 | -2.7623 |
| Novel01724 | 354.7320753 | 1.888349065 | -7.5535 |
| gene40818 | 166.4895759 | 1.529074057 | -6.7666 |
