# Supplementary material for: NtbHLH49, a jasmonate-regulated transcription factor, negatively regulates tobacco responses to Phytophthora nicotianae
Source: Front Plant Sci. 2022 Dec 6;13:1073856. doi: 10.3389/fpls.2022.1073856 (PMC9764443; doi:10.3389/fpls.2022.1073856)
Supplement: Supplementary file 5 [file DataSheet_1.docx]

**Supplementary Figure**

**NtbHLH49, a Jasmonate-Regulated Transcription Factor, Negatively Regulates Tobacco Responses to *Phytophthora nicotianae***

Wenjing Wang^1,#,*^, Jianhui Zhang^2,#^, Yi Cao^3^, Xingyou Yang^2^, Fenglong Wang^1^, Jinguang Yang^1^, Xiaoqiang Wang^1^

^1^Tobacco Research Institute, Chinese Academy of Agricultural Sciences, Qingdao 266101, China;

^2^Sichuan Tobacco Science Research Institute, Chengdu 610041, China;

^3^Academy of Guizhou Tobacco Sciences, Guiyang 550081, China.

^#^These authors contributed equally to this work.

^*^To whom correspondence should be addressed: E-mail: [wangwenjing@caas.cn](mailto:zhanghongbo@caas.cn).

**
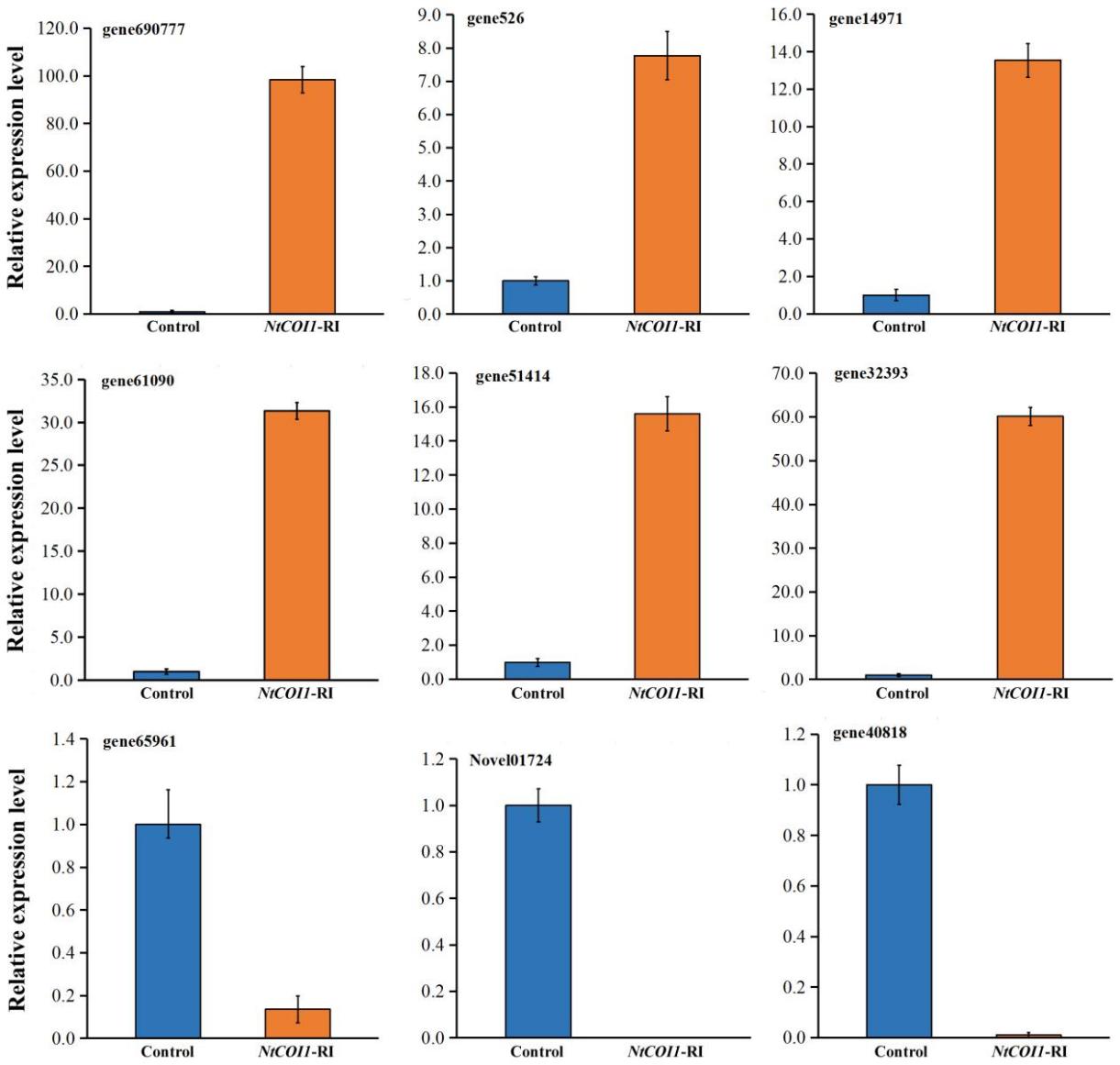
**

**FIGURE 1.** Relative expression of the genes selected for validation in control and *NtCOI1*-RI plants. Tobacco *Actin* gene was used as an internal control. The expression level of each gene in control was set as “1”.
